# BRCA1 and BRCA2 Gene Mutations in Reproductive-Aged Women With Polycystic Ovarian Syndrome

**DOI:** 10.7759/cureus.82496

**Published:** 2025-04-18

**Authors:** Soujanya Pujar, Neelamma Patil, Gurushantappa S Kadakol

**Affiliations:** 1 Obstetrics and Gynecological Nursing, Bharatiya Lingayat Development Educational Association (BLDEA) Shri B M Patil Institute of Nursing Sciences, Vijayapura, IND; 2 Obstetrics and Gynecology, BLDE (Deemed to be University) Shri B M Patil Medical College, Hospital and Research Centre, Vijayapura, IND; 3 Anatomy and Human Genetics, BLDE (Deemed to be University) Shri B M Patil Medical College, Hospital and Research Centre, Vijayapura, IND

**Keywords:** brca1 gene, brca2 gene, mutations, polycystic ovarian syndrome, risk factors

## Abstract

Introduction: Polycystic ovarian syndrome (PCOS) is a heterogeneous condition influenced by genetic, environmental, and lifestyle factors. The aim of this study was to evaluate the relationship between BRCA1 and BRCA2 gene mutations with PCOS and to determine the sociodemographic and clinical risk factors.

Aim and objective: This study seeks to analyze mutations in the BRCA1 and BRCA2 genes among women with PCOS in the Vijayapura region.

Methodology: In total, there were 96 individuals included (49 PCOS individuals and 47 healthy controls). Blood samples were collected for genetic analysis, and the anthropometry parameters, menstrual irregularities, and ultrasound findings were recorded. Genomic DNA was extracted to undergo polymerase chain reaction (PCR) followed by automated sequencing. Statistical analysis was performed using IBM SPSS Statistics for Windows, Version 20.0 (Released 2011; IBM Corp., Armonk, New York, United States), with a significance level set at p<0.05.

Results: Outcomes presented an association between rural residency, educational status, and consanguineous marriage, as well as homemaker and PCOS (p=0.001 for all). Abnormal menstruation and dysmenorrhea (p=0.001) occurred frequently (69.4%) in women with PCOS (89.8% had acne). BRCA1 mutations exhibited a significant association with PCOS (p=0.045), where 8.2% of cases expressed the rs1555600862 (C>G variant, a likely benign missense mutation).

Conclusion: PCOS women often have a hormonal imbalance and ovarian dysfunction. Detecting BRCA gene mutations in women with PCOS is very crucial because these mutations are linked to higher risks of breast and ovarian cancers. Early detection of the BRCA gene mutation status can provide clear, valuable insight in terms of the prevention of complications and treatment options.

## Introduction

Polycystic ovarian syndrome (PCOS) is a multifaceted endocrine disorder affecting women of reproductive age, characterized by hormonal imbalances, menstrual irregularities, and metabolic issues. Genetic factors are thought to play a role in the risk for PCOS [1]. BRCA1 and BRCA2 are tumor suppressor genes that, when inherited as a single copy of the mutated version, can lead to loss of function and, as a result, to the risk of breast and ovarian cancer [2]. The BRCA1 gene is located on chromosome 17q21.31, consisting of 22 exons, whereas the BRCA2 gene is located on the long arm of chromosome 13q12.3 with 27 exons [3,4]. PCOS is a heterogeneous condition that manifests as increased androgens and varying levels of hormones and metabolic abnormalities [5]. The predisposing factors, like genetic, hormonal, and metabolic, are involved in the association of PCOS and enhanced cancer risk towards ovarian and breast cancers. Among women with PCOS, obesity, decreased physical activity, and sedentary lifestyles, often associated with urbanization and high-fat diets, are the commonly observed clinical risk factors [6]. It is also influenced by age and geographical location, as well as by socioeconomic conditions and reproductive events [7]. Despite its significance, research on BRCA gene mutations specific to the South Indian population remains less. Despite its significance, there remains a notable gap in research exploring BRCA gene mutations specifically in the South Indian population.

## Materials and methods

This case-control study included 96 participants, consisting of 49 women with PCOS (cases) and 47 controls. The investigation took place at the Obstetrics and Gynecology Outpatient Department of BLDE (Deemed to be University), Shri B M Patil Medical College, Hospital and Research Centre, located in Vijayapura, Karnataka, India. The research was conducted in the Genetics Laboratory and the Centre for Advanced Medical Research (CAMR) from October 2022 to July 2023. Prior to the start of the study, ethical approval was secured from the institute's Institutional Ethical Committee (approval number: BLDE (DU)/IEC/584/2021-22). Informed consent was obtained by clearly explaining the study's purpose and procedures to the patients before gathering blood samples. The sample size was determined to ensure a mean with a confidence interval of 95%, assuming a one-to-one ratio of presence to absence.

Inclusion criteria

The research involved women between the ages of 18 and 35, who voluntarily agreed to participate.

Exclusion criteria

Individuals were not included if they had confirmed pregnancies, had other medical or surgical conditions, were utilizing contraceptive pills or hormone replacement therapy, or had a personal or family history of breast cancer.

Sample collection

**Figure 1:**
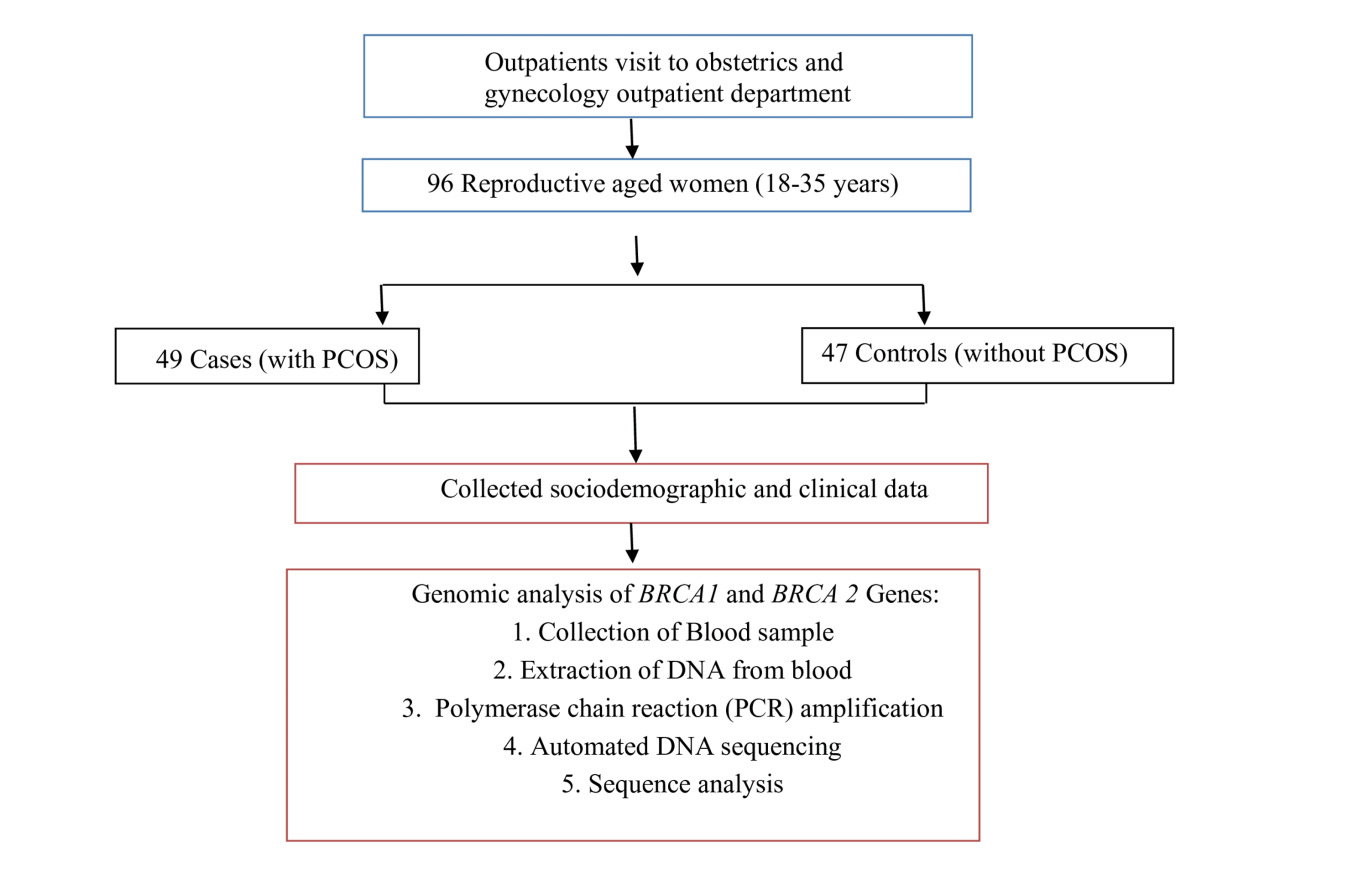
Flowchart of participant recruitment and steps of genetic testing PCOS: polycystic ovarian syndrome

The women with PCOS are recruited based on the Rotterdam criteria [6]. To compare the results, fertile women of the same age group were selected as controls. All participants underwent a detailed assessment with sociodemographic history, anthropometric measurements, and clinical examination. A total of 1 ml of blood was drawn from each participant using K2 EDTA vacutainers (Labtech Disposables, Ahmedabad, India). After collection, the samples were processed in the lab according to standard protocols and stored at -20°C for future research. The flowchart of participant recruitment and the steps of genetic testing are presented in Figure 1.

Extraction of DNA from blood

DNA extraction from blood utilized approximately 200 μl of blood samples in conjunction with a DNA isolation kit (NucleoSpin, Macherey-Nagel, Düren, Germany), carried out by a silica membrane-based approach. The Infinite 200 PRO plate reader (Tecan, Männedorf, Switzerland) was used to assess the quality and quantity of DNA. The designed sequences employed for targeting the BRCA1 and BRCA2 genes are presented in Table 1.

**Table 1:** Details of primers for BRCA1 and BRCA2 genes

No.	Gene name	Location	Primer (5’ sequence 3’)	Amplicon size	Annealing temperature
1	BRCA1	Exon 2: Forward	GAAGTTGTGTCCATTTTATAAACCTTT	293 bp	60°
2	BRCA1	Exon 2: Reverse	GTC TTT TCT TCC CTA GTA TGT		
3	BRCA2	Exon 12: Forward	AAG CGT GAG GGG ACA GA	230 bp	60°
4	BRCA2	Exon 12: Reverse	GAC CTT TCT CTC AGG CAT G		

Genomic DNA samples underwent standard polymerase chain reaction (PCR) procedures. The final reaction volume of 20 μl comprised 1 μl of DNA (with concentrations ranging from 20 ng/μl to 100 ng/μl), 10 μl of a pre-prepared master mix containing each deoxynucleotide triphosphate (dNTP) at 0.4 μl (5 pmol), forward and reverse primers at 0.4 μl each (5 pmol), 0.2 μl of Taq DNA polymerase (3 U/μl), and 4 μl of Taq buffer (5×) from Takara, Japan. The total volume was adjusted to 20 μl using nuclease-free water. These PCR reactions were conducted in a thermocycler (Veriti, Thermo Fisher Scientific, Waltham, Massachusetts, United States) under the following cycling conditions: an initial denaturation at 95°C for five minutes, followed by 35 cycles of denaturation at 95°C for 30 seconds, primer annealing at 56°C for 30 seconds, elongation at 72°C for one minute, and a final extension at 72°C for five minutes. The resulting products were analyzed via 2% agarose gel electrophoresis alongside a 100 bp DNA ladder to verify the PCR outcomes. After confirmation, all PCR products were subjected to automated DNA sequencing (ABI_3500xl), utilizing both forward and reverse primers for each sample. The electropherograms generated from the automated sequencing were evaluated for sequence quality using Sequence Analysis Software (ABI) (Applied Biosystems, Waltham, Massachusetts, United States).

Statistical analysis

Data analysis was performed using IBM SPSS Statistics for Windows, Version 20.0 (Released 2011; IBM Corp., Armonk, New York, United States). Descriptive statistics included means, standard deviations, counts, and percentages. For group comparisons, t-tests were used for normal data and Mann-Whitney U tests for non-normal data. Chi-squared tests were used for categorical variables. The confidence intervals (95% CI) and p-values were reported for each comparison, with significance set at p<0.05.

## Results

The study included 96 participants: 49 women with PCOS and 47 controls. Most participants were 21-25 years old in both groups. PCOS was significantly associated with marital status (p=0.004), with more married women in the PCOS group (75.5%) than in the control group (46.8%). Consanguineous marriages were more common in the PCOS group (53.3%) compared to controls (8.5%). Employment status differed significantly between groups (p=0.001). Most PCOS cases (67.3%) were homemakers, while controls were either students (57.4%) or employed (42.6%). Education levels also showed a significant association (p=0.001), with more PCOS cases (69.4%) having degrees compared to controls (59.6%). Table 2 presents the sociodemographic data and their correlation with PCOS.

**Table 2:** Sociodemographic data and their correlation with PCOS PG: postgraduate; PCOS: polycystic ovarian syndrome * indicates statistical significance, representing a p-value less than or equal to a predetermined significance level (usually 0.05 or 5%)

	Cases (n=49)	Percentage (%)	Controls (n=47)		Percentage (%)	Chi-squared test	P-value
Age in years							
<21	7	14.3	11		23.4	5.520	0.238
21-25	22	44.9	18		38.3		
26-30	13	26.5	7		14.9		
31-35	7	14.2	11		23.4		
Marital status							
Married	37	75.5	22		46.8	8.343	0.004*
Unmarried	12	24.5	25		53.2		
Type of marriage							
Consanguineous	24	49		4	8.5	29.695	0.001*
Non-consanguineous	21	42.9		18	38.3		
Employment							
Homemaker	33	67.3		0	0	48.716	0.001*
Student	11	22.4		27	57.4		
Working	5	10.2		20	42.6		
Education							
Secondary school	6	12.2		0	0	16.306	0.001*
High school	3	6.1		0	0		
Degree	34	69.4		28	59.6		
PG	6	12.2		19	40.4		

In the study, researchers observed that all cases had irregular menstruation cycles with a significant p-value (0.001). The majority of cases (59.2%) have painful menstruation compared to controls. Hirsutism (69.4%), acne (89.8%), and acanthosis nigricans (46.9%) were found more in cases compared to controls. Around 75.5% of cases have a family history of PCOS. Painful menstruation, acanthosis nigricans, and a family history of PCOS are significantly associated with PCOS. The details of clinical data and their association with PCOS are given in Table 3.

**Table 3:** Clinical data and their association with PCOS PCOS: polycystic ovarian syndrome * indicates statistical significance, representing a p-value less than or equal to a predetermined significance level (usually 0.05 or 5%)

Clinical parameter	PCOS cases n=49 (%)	Controls n=47 (%)	Chi-squared test	P-value
Menstrual cycles				
Irregular	49 (100)	0 (0)	96	0.001*
Regular	0 (0)	47 (100)		
Dysmenorrhea				
No	20 (40.8)	38 (80.9)	16.078	0.001*
Yes	29 (59.2)	9 (19.1)		
Hirsutism	34 (69.4)	4 (8.5)	37.1759	0.141
Acne	44 (89.8)	6 (12.8)	5.267	0.153
Acanthosis nigricans	23 (46.9)	2 (4.3)	77.085	0.001*
Family history of PCOS	37 (75.5)	2 (4.25)	50.4958	0.001*
Height in cm (mean±SD)	155.29±5.1	153.21±4.8	787.000	0.007
Weight in kg (mean±SD)	57.16±8.656	53.36±9.363	905.000	0.069
BMI (mean±SD)	23.6954±3.58729	22.7471±3.94177	1004.000	0.279

Out of 96 participants, comprising 49 women diagnosed with PCOS and 47 controls, only four patients showed mutations in the BRCA1 gene, while no controls had it, resulting in a significant association between the BRCA1 gene and PCOS (p=0.045). In contrast, 6.1% of cases and 6.38% of controls showed mutations, with no significant association between the BRCA2 gene and PCOS. The details of BRCA1 and BRCA2 gene mutations in PCOS patients and controls are given in Table 4.

**Table 4:** BRCA1 and BRCA2 gene mutations in PCOS patients and controls PCOS: polycystic ovarian syndrome * indicates statistical significance, representing a p-value less than or equal to a predetermined significance level (usually 0.05 or 5%)

	Cases (n=49)	Percentage (%)	Controls (n=47)	Percentage (%)	Chi-squared test	P-value
BRCA1 mutation						
Present	4	8.2	0	0	4.004	0.045*
Absent	45	91.8	47	100		
Total	49	100	47	100		
BRCA2 mutation						
Present	3	6.1	3	6.38	0.003	0.958
Absent	46	93.9	44	93.6		
Total	49	100	47	100		

Two mutations were found in the BRCA1 and BRCA2 genes among PCOS patients. A significant association was found between BRCA1 mutations and PCOS (p=0.045), with 8.2% of cases carrying the rs1555600862 (C>G), a likely benign missense variant. A heterozygous 5 prime UTR variant (c.-52 A>G, rs206118) in the BRCA2 gene was found in three cases as well as controls (p=0.958). No novel mutations were found in the BRCA1 and BRCA2 genes among PCOS patients. The mutation details of the BRCA1 and BRCA2 genes are given in Table 5.

**Table 5:** Mutation details of the BRCA1 and BRCA2 genes

Gene	Condition	gDNA position	cDNA position	aa position	Status	Genomic coordinates	Variant type	Phenotype/disease
BRCA1	Heterozygous	g.93965 C>G	c.78 C>G	p.I26M	rs1555600862	17q21.31 (41196312-41277381)	Missense variant	Likely benign
BRCA2	Heterozygous	g.5176 A>G	c.-52 A>G	NA	rs206118	13q12.3 (32,315,077-32,400,268)	5 prime UTR variant	Benign/uncertain significance

## Discussion

Considerable efforts have been made to investigate the genetic foundation of PCOS and its association with specific gene mutations. This South Indian cohort study focused on exploring the genetic association in PCOS women. Among 96 study participants, four women with PCOS were identified as having BRCA1 gene mutations, whereas no such mutations were detected in the control group. Specific variant observations were noted in exon 2 of the BRCA1 gene and exon 12 of the BRCA2 gene among women with PCOS, suggesting a potential role of BRCA gene mutations in the underlying mechanisms of PCOS.

This research enhances the understanding of sociodemographic, clinical, and genetic factors related to PCOS. The significant association between rural residence and PCOS (69.4% of cases; p=0.001) suggests that environmental factors may play a role in the disorder's development. Bhatti et al.'s study supports this finding by drawing attention to poor access to services and adverse lifestyle habits among rural women with PCOS [7].

The significant association between lower education and PCOS (p=0.001) highlights the role of healthy literacy in managing PCOS. Postgraduate qualifications were possessed by only 12.2% of PCOS women compared with 40.4% of controls, suggesting that limited access to information on health may limit the management of health. A parallel study found that only 32.4% of PCOS women completed their postgraduate education. Lower levels of education were significantly correlated with reduced health literacy (p=0.0001) and awareness of PCOS (p=0.0001) [8]. Enabling educational access empowers women with PCOS to manage their health proactively.

The correlation between marital status and PCOS (p=0.004) necessitates further exploration of cultural and environmental factors as potential triggers. The significant link between consanguinity and PCOS instances (0%; p=0.001) suggests that a specific gene may serve as a model for PCOS development. This result corroborates the earlier findings of Abedalthagafi and ElBardis, validating consanguinity as a recognized risk factor [9]. Interestingly, an increased prevalence of unhealthy habits, including poor sleep, inappropriate diet, and lack of exercise, was noted among the PCOS population. A correlation exists between unemployment and the role of a homemaker, specifically concerning the development of PCOS, where 67.3% of afflicted individuals were homemakers (p=0.001), which points toward an association between a sedentary lifestyle and PCOS. Further evidence from Vidya et al.'s research supports this, with their findings indicating that physical inactivity, an increased intake of calorie-dense foods, and reliance on labor-saving domestic appliances are primary factors contributing to the higher prevalence of PCOS [10].

Menstrual irregularities and other clinical parameters were identified as key symptom indicators of PCOS. It is widely acknowledged that a considerable majority (55.1%; p=0.001) of individuals experiencing uterine bleeding are likely to suffer from menstrual irregularities, such as infrequent or prolonged periods, in line with findings by Li et al., who identified menstrual dysfunction as a characteristic feature of PCOS [11]. The study conducted by Behzadfar et al. found that dysmenorrhea, which is a common symptom in women with PCOS, typically manifests as menstrual cramps [12].

The study further supported the high incidence of symptoms of androgen excess in patients with PCOS, including hirsutism (69.4%), acne (89.8%), and acanthosis nigricans (46.9%). The discovery aligns with a study by Ashraf et al., which indicates that hormonal imbalances are the common characteristics of PCOS and underscore the importance of the early detection of these symptoms to ensure accurate diagnosis and treatment [13].

Data from our region revealed that BRCA1 and BRCA2 gene variants among women with PCOS exhibited common mutations, with no newly discovered mutations identified in targeted regions screened in either cases or controls. The results indicate a significantly high positive association between the BRCA1 gene mutation and susceptibility to PCOS, suggesting a strong genetic bond. The studies conducted by Chen et al. also suggest that women with genetically programmed PCOS are more susceptible to the development of ER-positive breast cancer. The main point of BRCA1 mutations is relevant to both diseases and strategies for preventing breast cancer [14]. Conversely, Siddamalla et al. did not find a significant relationship between BRCA2 mutations and PCOS, aligning with previous research suggesting that BRCA2 is not a major factor in PCOS pathogenesis [15].

Limitations

This research involves a small sample size and focuses on a particular region of Southern India. Hence, the relationship between BRCA gene changes and hormones in PCOS women cannot be applied universally. Future research plans include expanding the sample size to further evaluate the impact of hormones and genes, as well as investigating the benefits of lifestyle modifications. The requirement for specialized laboratory equipment in genetic studies restricts its availability, particularly for women from middle and lower socioeconomic backgrounds.

## Conclusions

This study highlights a significant familial correlation between BRCA1 mutations and susceptibility to PCOS. It further shows that ovarian dysfunction, which is more pronounced than the influence of BRCA2 mutations, is largely influenced by BRCA1 polymorphisms. Although no novel mutations were found, the identified BRCA1 variants suggest a potential genetic link to PCOS. Larger studies are needed to explore the functional impact of these findings. For a complete understanding of the implications of these observations, larger-scale functional analyses and observational studies are needed. Future studies will also need to investigate further the genetic basis of PCOS, especially epigenetic changes and gene-environment interactions, to understand their impact on ovarian function in women with PCOS. Such insights could contribute to better risk assessment and personalized management strategies for PCOS women, possibly including BRCA screening as a part of extended genetic assessment.
